# Role of chemical composition and redox modification of poorly soluble nanomaterials on their ability to enhance allergic airway sensitisation in mice

**DOI:** 10.1186/s12989-019-0320-6

**Published:** 2019-10-28

**Authors:** Susan Dekkers, James G. Wagner, Rob J. Vandebriel, Elyse A. Eldridge, Selina V. Y. Tang, Mark R. Miller, Isabella Römer, Wim H. de Jong, Jack R. Harkema, Flemming R. Cassee

**Affiliations:** 1 National Institute for Public Health and the Environment (RIVM), P.O.Box 1, 3720 BA Bilthoven, The Netherlands; 2 Department of Pathobiology and Diagnostic Investigation, Michigan State University, East Lansing, MI USA; 3 Promethean Particles Ltd, Nottingham, UK; 4 Centre for Cardiovascular Science, University of Edinburgh, Edinburgh, UK; 5 School of Geography, Earth and Environmental Sciences, University of Birmingham, Birmingham, UK; 6 Institute for Risk Assessment Sciences, Utrecht University, Utrecht, the Netherlands

**Keywords:** Nanomaterials, Cobalt oxide, Cerium dioxide, Redox activity, Adjuvant, Allergy, Poorly soluble

## Abstract

**Background:**

Engineered nanoparticles (NPs) have been shown to enhance allergic airways disease in mice. However, the influence of the different physicochemical properties of these particles on their adjuvant properties is largely unknown. Here we investigate the effects of chemical composition and redox activity of poorly soluble NPs on their adjuvant potency in a mouse model of airway hypersensitivity.

**Results:**

NPs of roughly similar sizes with different chemical composition and redox activity, including CeO_2_, Zr-doped CeO_2_, Co_3_O_4_, Fe-doped Co_3_O_4_(using Fe_2_O_3_ or Fe_3_O_4_) and TiO_2_ NPs, all showed adjuvant activity. OVA induced immune responses following intranasal exposure of BALB/c mice to 0.02% OVA in combination with 200 μg NPs during sensitization (on day 1, 3, 6 and 8) and 0.5% OVA only during challenge (day 22, 23 and 24) were more pronounced compared to the same OVA treatment regime without NPs. Changes in OVA-specific IgE and IgG1 plasma levels, differential cell count and cytokines in bronchoalveolar lavage fluid (BALF), and histopathological detection of mucosa cell metaplasia and eosinophil density in the conducting airways were observed. Adjuvant activity of the CeO_2_ NPs was primarily mediated via the Th2 response, while that of the Co_3_O_4_ NPs was characterised by no or less marked increases in IgE plasma levels, BALF IL-4 and IL-5 concentrations and percentages of eosinophils in BALF and more pronounced increases in BALF IL-6 concentrations and percentages of lymphocytes in BALF. Co-exposure to Co_3_O_4_ NPs with OVA and subsequent OVA challenge also induced perivascular and peribronchiolar lymphoid cell accumulation and formation of ectopic lymphoid tissue in lungs. Responses to OVA combined with various NPs were not affected by the amount of doping or redox activity of the NPs.

**Conclusions:**

The findings indicate that chemical composition of NPs influences both the relative potency of NPs to exacerbate allergic airway sensitization and the type of immune response. However, no relation between the acellular redox activity and the observed adjuvant activity of the different NPs was found. Further research is needed to pinpoint the precise physiological properties of NPs and biological mechanisms determining adjuvant activity in order to facilitate a safe-by-design approach to NP development.

## Background

Engineered nanoparticles (NPs) are under increasing development for a wide-range of applications, however, their potential for toxicity still remains poorly understood. Identifying which physicochemical properties of NPs affect the potential toxicological effects will therefore facilitate predictive risk assessment, grouping and safe-by-design of NPs [1–4].

The relative large surface-to-volume ratio and surface reactivity of NPs are, for example, known to increase reactive oxygen species (ROS) generation [5, 6]. As a consequence, inhalation of NPs may lead to greater levels of cellular oxidative stress and, subsequently inflammation in the respiratory system, than their larger counterparts. In addition, existing inflammation (e.g. from asthma) can also be exacerbated by NP exposure [7, 8]. Nano-sized silica and titanium dioxide particles have been shown to enhance allergic airways sensitisation in mice [9, 10], however, there is limited understanding of the different physicochemical properties of NPs which influence their adjuvant properties. Previous studies on the influence of NPs on allergic airway sensitisation initially focussed on the role of the dendritic cell and T-cell interaction. Increases in the proliferation, maturation and/or differentiation of dendritic cells and T-cells have been observed after *in vitro *exposure to NPs and after *in vivo *exposure to NPs during the sensitisation phase [7]. Molecular interactions that drive these cellular responses have been suggested to involve increases in reactive oxygen species (ROS) caused by NPs with an oxidative surface chemistry, inflammasome activation, cellular injury and the induction of dendritic cell simulating cytokines or chemokines by epithelial cells (either direct or via oxidative stress) [7].

Nano-sized metal oxides are known to facilitate the formation of ROS by depleting electrons from cellular redox species (cellular components able to release electrons) or by serving as catalysts in ROS production through Fenton reactions or Haber-Weiss cycle reactions [11]. Additionally, NPs can stimulate free radical generation from cellular enzymes. Furthermore, biological fluids (e.g. epithelial lining fluid) and cells also contain antioxidants and numerous compensatory mechanisms, thus the relationship between ROS induction and toxicity is complex.

While there are clear associations between ROS generation and the toxicity of nanomaterials [11–13], the specific role of the redox activity of nanomaterials in the generation of ROS is difficult to investigate, since changing the redox activity of nanomaterials usually also changes other properties (such as the chemical composition or size). In a recent study, we applied chemical doping (intentional substitution of one element by another while maintaining the lattice structure and arrangement) of cerium dioxide to specifically investigate the influence of NP redox activity on ROS formation, and associated induction of oxidative stress responses in mice in vivo [14]. Different quantities of zirconium (Zr) were incorporated into the crystalline structure of the cerium dioxide nanoparticles (CeO_2_ NPs) to increase the antioxidant potential. However, Zr-doping of CeO_2_ NPs had limited effect on the inflammatory responses after inhalation in otherwise healthy mice.

Metal oxides with a conduction band energy (Ec) level that overlaps with the cellular redox potential (− 4.12 to − 4.84 eV) have been shown to have the ability to induce oxygen radicals, oxidative stress, and acute pulmonary inflammation [15]. Therefore, Co_3_O_4_, a metal oxide that (unlike CeO_2_) has an Ec level overlapping with the cellular redox potential was doped using Fe_2_O_3_ or Fe_3_O_4_ (Fe oxides themselves do not overlap with the cellular redox potential). Here we present an airway exposure study in a mouse model for airway allergy using the following poorly soluble NPs differing in redox potential but with similar sizes: a) Co_3_O_4_ NPs doped with different amounts of Fe, b) CeO_2_ NPs doped with different amounts of Zr, c) CeO_2_ NM212 NPs (for comparison to other *in vivo *studies) and d) TiO_2_ NPs (as a positive control [10]). We hypothesise that Co_3_O_4_ NPs will have a greater ability to enhance allergic airway sensitisation compared to (Zr-doped) CeO_2_ NPs, but that these effects of Co_3_O_4_ NPs can be reduced by Fe-doping. The outcome of this work will facilitate the assessment of the potential hazard of NPs to enhance allergic airway sensitisation and inform efforts to group NPs or to apply a safe-by-design approach in their development.

## Methods

### Experimental protocol

**Fig. 1:**
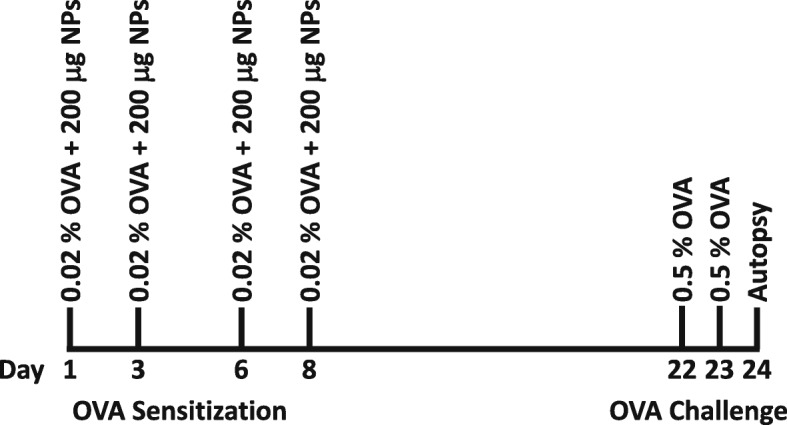
Schematic overview of the study design

Studies were designed to test the enhancement of allergic sensitization by airway co-exposure (intranasal instillation) of an experimental allergen (OVA) with a range of NPs that differ in chemical composition (Fig. 1). Animals were divided into 10 test groups and 3 control groups, each with 6 mice except OVA-control (*n* = 8) (see Table 1). On days 1, 3, 6 and 8 the mice were sensitized with 0.02% OVA in combination with 200 μg TiO_2_ NPs (positive controls), 0.02% OVA in combination with 200 µg of one of the other NPs (test groups), 0.02% OVA in PBS (OVA control) or PBS alone (PBS control). On days 22 and 23 all mice were challenged with 30 μl of 0.5% OVA in PBS. The animals were sacrificed 24 h after the last intranasal challenge (day 24).

**Table 1:** Overview of the study groups

Group	n	Day 1, 3, 6 and 8	Day 22 and 23	Day 24
1	6	PBS controls	OVA	necropsy
2	8	OVA controls	OVA	necropsy
3	6	TiO_2_ + OVA (NP positive controls)	OVA	necropsy
4	6	CeO_2_ NM212 + OVA	OVA	necropsy
5	6	Co_3_O_4_(0% Fe_2_O_3_) + OVA	OVA	necropsy
6	6	Co_3_O_4_(25% Fe_2_O_3_) + OVA	OVA	necropsy
7	6	Co_3_O_4_(75% Fe_2_O_3_) + OVA	OVA	necropsy
8	6	Co_3_O_4_(0% Fe_3_O_4_) + OVA	OVA	necropsy
9	6	Co_3_O_4_(25% Fe_3_O_4_) + OVA	OVA	necropsy
10	6	Co_3_O_4_(75% Fe_3_O_4_) + OVA	OVA	necropsy
11	6	CeO_2_(0% Zr) + OVA	OVA	necropsy
12	6	CeO_2_(27% Zr) + OVA	OVA	necropsy
13	6	CeO_2_(78% Zr) + OVA	OVA	necropsy

A dose of 200 μg of NPs was chosen based on our results in previous studies (with dose-ranges between 100 and 400 μg) using silica [9] and titanium dioxide [10]. The mouse model was developed with low concentrations of OVA during sensitization (0.02%) that produce minimal allergic responses with challenge, but can be dramatically enhanced with co-sensitization with an adjuvant such as particulate matter [9].

No experimental groups were included to measure the effects of exposure to NPs without OVA during the sensitisation phase, since previous studies showed that the allergic sensitisation response can only be enhanced via co-exposure of the allergen and SiO_2_ or TiO_2_ NPs, and not by exposure to SiO_2_ and TiO_2_ NP NPs alone [9, 10]. Although other studies have shown that separate administration of the allergen and NPs may also lead to adjuvant effects [16], in this study we have chosen to administer OVA and NPs in one suspension, in alignment with our previous studies.

### Nanomaterial production and characterization

TiO_2_ NPs (NO-0058-HP) were obtained from Ionic Liquids Technologies GmbH, Germany. These uncoated anatase TiO_2_ NPs were the same NPs as used on our previous *in vivo *study [10]. The OECD representative manufactured nanomaterial CeO_2_ (NM212) was obtained from Umicore, Belgium. The other CeO_2_ NPs, Zr-doped CeO_2_ NPs, Co_3_O_4_ NPs and Fe-doped Co_3_O_4_ NPs were produced using supercritical water hydrothermal synthesis [17, 18]. Briefly, H_2_O was pumped through a pre-heating coil (~ 400 °C), brought into contact with a concurrently flowing solution of metal salts at room temperature (RT), while maintaining the flow rates, temperature and the pressure constant at 240 bar. The mixture was cooled immediately after the mixing point and passed through a back pressure regulator to decrease the pressure back down to ambient conditions. Solids in the aqueous suspensions were washed by centrifuging and re-dispersing in clean MilliQ water 3 times. The particles were characterised in suspension or, where solid particles were required, the suspensions were oven dried (~ 100 °C). For the synthesis of the Fe-doped Co_3_O_4_ NPs, different ratios of cobalt and iron salts were used as precursors. Two different iron salts (iron (III) nitrate nonahydrate and ammonium iron (III) citrate) were used, resulting in two different series of NPs, with Fe_2_O_3_ or Fe_3_O_4_ as a precursor, respectively. These different series (Co_3_O_4_(x% Fe_2_O_3_) NPs and Co_3_O_4_(x% Fe_3_O_4_) NPs) have different degrees of crystallinity, aggregation, Co/Fe ratio and spatial distributions of the metals in the NPs [18].

The particle size in ultra-pure water was measured with disc centrifuge. The particle size in cell culture medium (RPMI 1640, Gibco) with 25 mM Hepes and 2 mM L-Glutamine, 1% Penicilline-Streptomycine (Pen/Strep 10,000 U/mL, Glibco) and 10% heat inactivated Fetal Calf Serum (FCS, Glibco), was measured with dynamic light scattering (DLS). The redox activity was tested with electron paramagnetic resonance (EPR) using a cell free system with a superoxide-selective spin-trap (tempone-H) to detect the generation of superoxide production and the spin trap DMPO [19] in combination with H_2_O_2_ and CuSO_4_ to analyse the capacity to scavenge hydroxyl free radicals. For the superoxide-selective spin-trap nanoparticle suspensions (0.56 mg/mL) were prepared and incubated with the spin-trap, Tempone-H (1 mM) immediately before the initial measurement. Samples were kept at 37 °C throughout and measurements were taken with an X-band EPR spectrometer (Magnettech MS-200, Berlin, Germany) at 60 min by drawing 50 μL of sample into a capillary tube (Scientific Laboratory Ltd., Coatbridge, UK) and sealing with a plug of soft sealant (Cristaseal, VWR International, UK). For the DMPO spin trap a sample was made of 12.5 μL nanoparticle suspension (1.28 mg/mL), 12.5 μL CuSO_4_ (20 μM), 25 μL H_2_O_2_ (0.5 M) and 50 μL DMPO (0.05 M). This sample was incubated in a shaker water bath at 37 °C for 15 min at 100 rpm, vortexed and taken up in a capillary tube, which was then sealed at the bottom with haematocrit and measured with the ESR Spectroscope (Miniscope MS 400; MT MagnetTech GmbH). All NPs were tested for endotoxin contamination using either a Limulus Amoebocyte Lysate Assay (chromogenic kinetic LAL assay) or LC-MS/MS determination [20].

### Animals

Female BALB/c mice (~ 20 g body weight; 6–8 weeks old) were obtained from Charles River (Portage, MI). Mice were maintained at the Michigan State University (MSU) animal housing facilities at room temperature (21 °C–24 °C) and relative humidity of 45–70%, with a 12 h light/dark cycle. MSU is AAALAC accredited and all animal procedures & experimental protocols were approved by the MSU Institutional Animal Care and Use Committee.

### Intranasal instillation

Mice were lightly anesthetized for 1–2 min with 4% isoflurane in oxygen using an isoflurane vaporizer. Cradling the mouse in an upright position, the thumb was used to support the lower jaw and position the nose of the mouse so it was easily accessible. A volume of 30 μL was delivered to the tip of the nares. Once the instillate was fully inhaled, the mouse was placed back in its cage and monitored until conscious with normal respirations.

### Necropsy, lavage collection and tissue preparation

Mice were anesthetized with an intraperitoneal injection of sodium pentobarbital (60 mg/kg body weight). A midline laparotomy was performed and approximately 0.5 mL of blood was drawn from the vena cava and collected in heparinized tubes (BD Microtainer, Franklin Lakes, NJ) for separation of plasma. The plasma was stored at − 80 °C for later biochemical analysis (OVA-specific IgE and IgG1). Animals were exsanguinated and the trachea was exposed and cannulated, and the heart and lungs were excised en bloc. A volume of 0.8 mL sterile saline was instilled through the tracheal cannula and withdrawn to recover bronchoalveolar lavage fluid (BALF). A second intratracheal saline lavage was performed and the collected BALF was combined with the first sample for analysis.

After the BALF was collected, the left lung lobe was intratracheally fixed with neutral-buffered formalin at a constant pressure (30 cm H_2_O) for 2 h and then submersed in the same fixative for > 24 h until further tissue processing for light microscopy. Two sections were excised at the level of the 5^th^ and 11^th^ airway along the main axial airway (G5 and G11), to sample proximal and distal bronchiolar airways, respectively [21]. Tissue was embedded in paraffin and 5- to 6-μm-thick sections were cut from the anterior surface. Lung sections were stained with hematoxylin and eosin (H&E) for routine light microscopic examination and with Alcian Blue (pH 2.5)/Periodic Acid–Schiff (AB/PAS) for identification of intraepithelial neutral and acidic mucosubstances in pulmonary bronchiolar epithelium. To detect eosinophils, slides were immunostained using a polyclonal rabbit antibody directed against murine eosinophil-specific major basic protein (MBP; 1:500; Mayo Clinic, AZ). Accumulations of B-lymphocytes in lung tissues were detected by immunohistochemical staining with rat anti-CD45R monoclonal (1:600; Becton Dickinson, Franklin Lakes, NJ, catalog # 550286).

### OVA-specific IgE and IgG1 in plasma

Plasma was separated from blood and analysed for OVA-specific IgE and IgG1 using an ELISA kit (Cayman, Ann Arbor, MI) according to the manufacturer’s instructions. The plasma samples were diluted 8 times for the IgE and 1000 times for the IgG1 analysis.

### BALF analyses

The total number of cells in the BALF was determined using a hemocytometer. Cytological slides were prepared by centrifugation at 40 x g for 10 min at 20 °C using a Shandon cytospin 3 (Shandon Scientific, PA) and stained with Diff-Quick (Dade Behring, DE). Differential cell counts for neutrophils, eosinophils, macrophages/monocytes, and lymphocytes were assessed from a total of at least 200 cells. The remaining BALF was centrifuged at 240 x g for 15 min at 4 °C and the supernatant was collected and stored at − 80 °C for subsequent measurement of inflammatory cytokines (IFNγ, IL-4, IL-5, IL-1β, IL-6, IL-13, IL-17 and TNFα) using a Luminex-kit (Millipore, MA).

### Lung morphometry

Histologic slides with lung tissue sections were scanned and digitized with a slide scanner (VS110, Olympus America, Center Valley, PA), and evaluated using stereological methods with newCAST software (VisioPharm, Hoersholm, Denmark). For quantification of major basic protein (MBP)-positive eosinophils, digitized images of the lung were selected as regions of interest and 40% of the lung tissue was captured at 400x magnification by systematic random sampling. Percentage of MBP-positive cells in the total lung tissue and in three discrete regions of 1) the parenchyma (alveoli and alveolar ducts), 2) perivascular and peribronchial interstitial spaces, and 3) other regions (inside blood vessels, bronchiole airspaces and pleura), were estimated using Stepanizer stereology software with a point grid by dividing the number of points hitting areas positive for MPB (*a*_*(p)positive*_) by the total number of points falling on all lung tissue (MBP-positive and –negative; *a*_*(p)reference tissue*_). For each region, percent density of eosinophils was calculated with the equation:

$$ Eosinophil\ Density\ \left(\%\right)=\frac{\left( No. positive\ cell s\right)\times {a}_{(p) positive\ cell}}{\left( No. reference\ tissue\right)\times {a}_{(p) reference\ tissue}}\times 100 $$

For quantification of CD45R-positive staining cells (the B-lymphocyte density), digitized images of G5 and G11 lung sections were selected as regions of interest and 40% of the lung tissue was captured at 400x magnification by systematic random sampling. Percentage of CD45R-positive cells in the perivascular and peribronchial interstitial spaces were estimated using Stepanizer stereology software using a point grid by dividing the number of points hitting areas positive for CD45R (*a*_*(p)positive*_) by the total number of points falling on all lung tissue (CD45R-positive and –negative; *a*_*(p)reference tissue*_). For each region, percent density of B-lymphocytes was calculated with the equation:

$$ B- lymphocyte\ Density\ \left(\%\right)=\frac{\left( No. positive\ cell s\right)\times {a}_{(p) positive\ cell}}{\left( No. reference\ tissue\right)\times {a}_{(p) reference\ tissue}}\times 100 $$

For quantification of AB/PAS-positive mucosubstances in the bronchiolar epithelium, all bronchiolar epithelium lining the main axial airway was selected and captured at 400x magnification. A point intercept grid was placed over the sampled images to estimate the density of mucosubstances per basal lamina. The number of points hitting AB/PAS-positive mucosubstances (*P*_*m*_) was counted. The density of AB/PAS-positive mucosubstances (V̂_m_) was estimated by multiplying the total number of *P*_*m*_ by the area/point (*a/p*) and dividing them by the number of points hitting the reference space (*n*) as shown in the equation.

$$ {\hat{V}}_m=\frac{\sum {P}_m\times a/p}{n} $$

The surface density of the basal lamina (Ŝ_BL_) in the selected images was estimated by counting the number of intercepts (*I*) of the line probe with the basal lamina of the lateral wall divided by the length per point (*l/p*) and the number of points falling on the reference space (*n*) as described:

$$ {\hat{S}}_{BL}=\frac{2\times \sum I}{l/p\times n} $$

The positive density per basal lamina of the bronchiolar epithelium was then estimated by dividing V̂_m_ by Ŝ_BL_.

### Statistical analyses

GraphPad Prism v7.00 (GraphPad Software, San Diego, California, USA) was used to analyse the data. All data are depicted as group means ± standard deviation (SD). First, outliers were identified with Grubbs’ test (alpha = 0.05) and removed. The Shapiro-Wilk test and the Brown-Forsythe test were used to test for normality and equal variances, respectively. Differences between the OVA control group and a) the other control groups (PBS or TiO_2_ NP exposed), b) the CeO_2_ NP exposed groups, c) the Co_3_O_4_(x% Fe_2_O_3_) NP exposed groups and d) the Co_3_O_4_(x% Fe_3_O_4_) NPs exposed groups were analysed using a one-way analysis of variance (ANOVA), followed by a Dunnett’s post-hoc multiple comparisons test comparing groups exposed to OVA with NPs to groups exposed to OVA alone. For all statistical analyses, a *p*-value of ≤ 0.05 was considered statistically significant.

If the *p*-value of the Shapiro-Wilk or Brown-Forsythe test was ≤ 0.05, the data were log-normally transformed and again tested for normality and equal variances. If the *p*-values of the Shapiro-Wilk and Brown-Forsythe tests of the log-normally transformed data were > 0.05, a one-way ANOVA followed by a Dunnett’s post-hoc multiple comparisons test was performed on the log-normally transformed data. If the *p*-values of the Shapiro-Wilk test were still ≤ 0.05, a non-parametric (Kruskal Wallis) ANOVA was performed on the non-transformed data followed by a Dunn’s post-hoc multiple comparisons test.

## Results

### Nanomaterial characterization

**Fig. 2:**
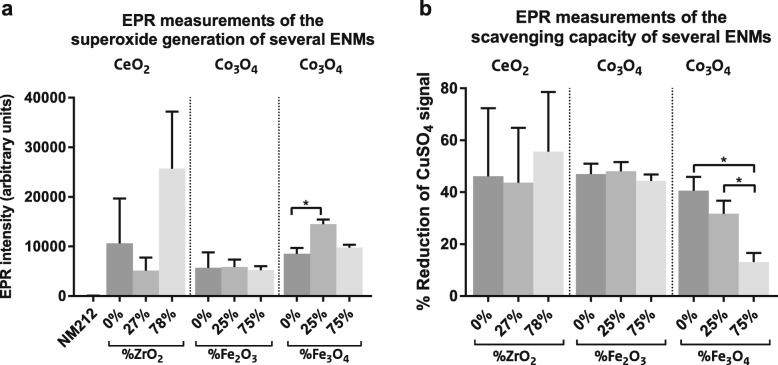
Reactive oxygen species (ROS) generation and scavenging capacity of NPs. Superoxide generation of NPs measured in a cell free system by electron paramagnetic resonance (EPR) using Tempone-H (**a**). The EPR signal of the Co_3_O_4_(25% Fe_3_O_4_) NPs was statistically significantly higher than the Co_3_O_4_(0% Fe_3_O_4_) NPs (*n* = 4) indicating a larger capacity to generate ROS. Scavenging capacity of several NPs expressed as the percentage reduction of the EPR signal of CuSO_4_ and NPs compared to CuSO_4_ alone, using a cell free system with a 5,5-dimethyl-1-pyrroline N-oxide (DMPO) spin trap in combination with H_2_O_2_ (**b**). The percentage reduction of the CuSO_4_ signal by the Co_3_O_4_(75% Fe_3_O_4_) NPs was significantly lower than that of the Co_3_O_4_(0 and 25% Fe_3_O_4_) NPs (*n* = 3), indicating a lower scavenging capacity of ROS

Fe-doping had small, but not statistically significant, effects on NP size in suspension (Table 2). Other characteristics, such as shape and crystalline structure, did not significantly change with increasing amounts of doping. In Fig. 2, the redox activity of the NPs as measured in a cell free system by EPR using two different spin-traps are shown. EPR analysis using tempone-H demonstrated an increased capacity of Co_3_O_4_(25% Fe_3_O_4_) NPs to generate reactive superoxide free radicals compared to Co_3_O_4_(0 and 75% Fe_3_O_4_) NPs. Increasing amounts of Fe-doping using Fe_3_O_4_ led a significant decrease in scavenging capacity of Co_3_O_4_ NPs. Co_3_O_4_(0, 25 and 75% Fe_2_O_3_) NPs had a similar ROS generation and scavenging capacity (Fig. 2). Endotoxin levels were below the recommended endotoxin limits for preclinical research in animal models (5 EU/kg bw/day or 36 EU/mL for mice with a body weight of 30 g and a daily dose of 100 μL) [22].

**Table 2:** Physicochemical characteristics of the nanoparticles

	Primary particle size (Mean ± SD) (nm)	Particle diameter in water (Median) (nm)	Particle diameter in cell culture medium + 10% foetal calf serum (Mean ± SD) (arbitrary units)
Measured with →	STEM	Disc Centrifuge (128 μg/mL)	DLS (128 μg/mL)
Anatase TiO_2_	10-15^a^	121^b^	–
CeO_2_ NM212	17.1 ± 10.9	–	428 ± 11.7
CeO_2_(0% Zr)	4.7 ± 1.4	39	288 ± 4.8
CeO_2_(27% Zr)	4.6 ± 1.4	40	176 ± 4.0
CeO_2_(78% Zr)	4.7 ± 1.4	41	415 ± 27.6
Co_3_O_4_(0% Fe_2_O_3_)	17.5 ± 15.0	42	176 ± 2.9
Co_3_O_4_(25% Fe_2_O_3_)	10.6 ± 3.6	62	303 ± 6.7
Co_3_O_4_(75% Fe_2_O_3_)	8.6 ± 1.7	39	373 ± 9.9
Co_3_O_4_(0% Fe_3_O_4_)	18.7 ± 11.2	44	160 ± 1.3
Co_3_O_4_(25% Fe_3_O_4_)	13.0 ± 5.2	61	828 ± 3.8
Co_3_O_4_(75% Fe_3_O_4_)	10.2 ± 6.2	69	1165 ± 66.5

^a^Information provided by the manufacturer instead of measured with STEM; ^b^ Particles size as measured in our previous study with Nanoparticle Tracking Analysis instead of Disc Centrifuge; *SD* Standard Deviation, *STEM* Scanning Transmission Electron Microscopy, *DLS* Dynamic Light Scattering, – = no data available

### OVA-specific IgE and IgG1 in plasma

**Fig. 3:**
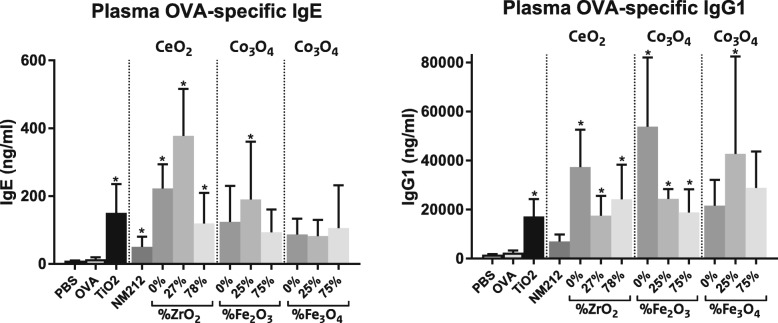
Concentration of OVA-specific IgE and IgG1 in plasma. Mean ± SD, *n* = 6 except OVA controls where *n* = 8, * = statistically significant different from OVA controls (*p* < 0.05)

OVA-specific IgE and IgG1 antibodies in plasma, indicating an OVA-specific immune response, were measured using an ELISA kit. OVA sensitization (0.02% OVA) and challenge (0.5% OVA) caused minimal, non-significant increases in plasma OVA-specific IgE and IgG1 compared to non-sensitized mice (phosphate-buffered saline (PBS) treated controls). Co-sensitization with NPs further increased the plasma OVA-specific IgE or IgG1 concentrations for all NPs. For all NPs, except for Co_3_O_4_(0 and 75% Fe_3_O_4_) NPs, the OVA-specific IgE and/or the IgG1 concentration was statistically significantly increased compared to OVA alone (Fig. 3).

### BALF analyses

#### BALF total cell count

**Fig. 4:**
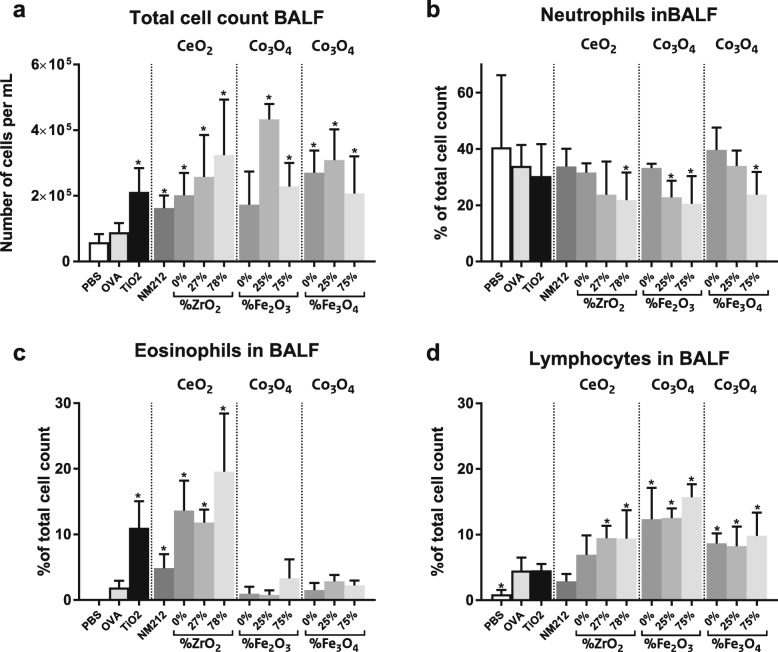
Differential cell counts in bronchoalveolar lavage fluid (BALF). Total cell count (**a**) and percentage of neutrophils (**b**), eosinophils (**c**) and lymphocytes (**d**) in the BALF. Mean ± SD, *n* = 6 except OVA where *n* = 8, * = statistically significant different from OVA controls (*p* < 0.05)

The total and differential cell counts were determined using a hemocytometer and analysis of cytospin prepared slides. All mice sensitized with NP plus OVA showed a significant increase in total BALF cells compared to the OVA controls, indicating an increased inflammatory response, except for animals exposed to Co_3_O_4_(0% Fe_2_O_3_) NPs (*p* = 0.18; see Fig. 4a). For CeO_2_ NPs the total cell count increased with increasing amounts of Zr doping.

#### Neutrophils

An increase in neutrophils in the BALF is indicative for a non-allergic, acute inflammatory response. No statistically significant differences between the percentage of neutrophils of the OVA controls and that of OVA plus NP exposed animals were observed, except for a decreased percentage of neutrophils in mice exposed to OVA plus CeO_2_(78% Zr) NPs, Co_3_O_4_(25 and 75% Fe_2_O_3_) NPs and Co_3_O_4_(75% Fe_3_O_4_) NPs. No major differences were observed between the different types of NPs (see Fig. 4b). The percentage of neutrophils decreased with the amount of doping for Zr-doped CeO_2_ NPs and Fe-doped Co_3_O_4_ NPs (using both Fe_2_O_3_ and Fe_3_O_4_).

#### Eosinophils and lymphocytes

The animals exposed to OVA plus CeO_2_ NPs showed a statistically significant increase in the percentage of eosinophils compared to the OVA controls (Fig. 4c), which is a typical feature of allergic asthma. For CeO_2_(27 and 78% Zr) NPs there was also a statistically significant increase in the percentage of lymphocytes (Fig. 4d), indicative for a chronic inflammatory response. Fe-doped Co_3_O_4_ NPs (using both Fe_2_O_3_ and Fe_3_O_4_) caused a statistically significant increase in the percentage of lymphocytes only, and not of eosinophils.

#### Monocytes

No major differences in the percentage of monocytes were observed between mice sensitized with NP plus OVA compared to the OVA controls (data not shown). More detailed data, including the absolute differential cell counts, can be found in Additional file 1.

#### BALF cytokines

**Fig. 5:**
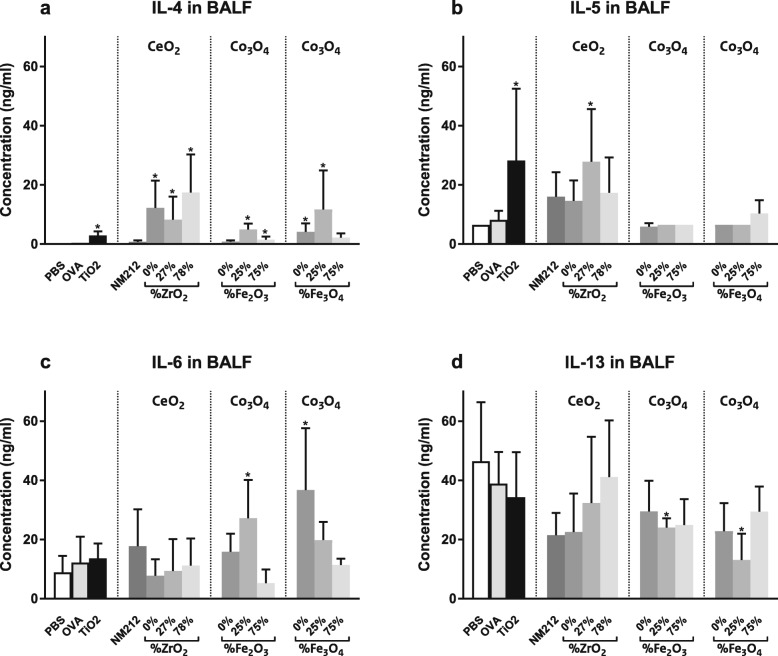
Cytokines in bronchoalveolar lavage fluid (BALF). Concentration of IL-4 (**a**), IL-5 (**b**), IL-6 (**c**) and IL-13 (**d**), in the BALF. Mean ± SD, *n* = 6 except OVA where *n* = 8, * = statistically significant different from OVA controls (*p* < 0.05)

The concentrations of several inflammatory cytokines in the BALF were determined in using a Luminex-kit specific for the various cytokines evaluated. Animals exposed to OVA plus CeO_2_ NPs showed increased concentrations of the Th2 cytokines IL-4 and IL-5 (but not IL-6 or IL-13) in the BALF compared to the OVA controls. Animals exposed to OVA plus most undoped and Fe-doped Co_3_O_4_ NPs showed increased concentrations of IL-6 compared to the OVA controls. All animals, including the controls (exposed to PBS, OVA and OVA plus TiO_2_ NPs during sensitization) had detectable concentrations of IL-13 (20–50 ng/mL) but these were not significantly different from one another (see Fig. 5d).

The concentrations of the other cytokines (IFNγ, IL-1β, IL-17 and TNFα) measured in BALF were either below the limits of detection or showed no significant changes in the OVA plus NP exposed groups compared to the OVA controls. More detailed data can be found in Additional file 2.

### Lung morphology

#### Mucous cell metaplasia

**Fig. 6:**
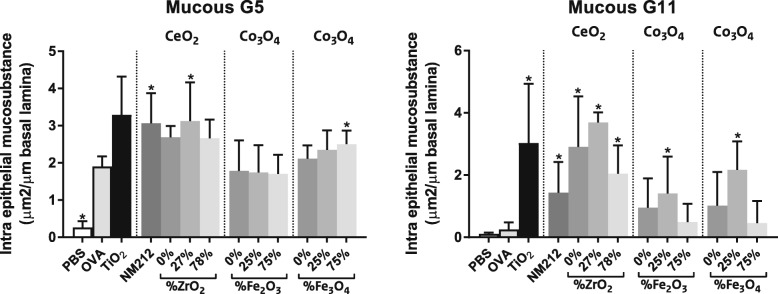
Mucous cell metaplasia. Quantification of the increase in intraepithelial mucosubstance in the epithelium lining the main proximal axial airway (G5; left) and distal axial airway (G11; right) of the left lung lobe. Mean ± SD, *n* = 6 except OVA where *n* = 8, * = statistically significant different from OVA controls (*p* < 0.05)

For quantification of epithelial mucous, lung tissue sections were stained with Alcian Blue/Periodic Acid Schiff (AB/PAS), a marker for neutral and acidic mucosubstances. Increased epithelial mucous production is a characteristic feature of allergic airway disease,

Sensitization and challenge with OVA caused a significant accumulation of intraepithelial mucosubstances (mucous cell metaplasia) in the epithelium lining the main proximal axial airways (G5), which was further enhanced by co-administration of OVA with TiO_2_ NPs (positive control). All CeO_2_ NPs enhanced mucosubstance accumulation; this was statistically significant for CeO_2_ NM212 and CeO_2_ with 27%, but not 78%, Zr doping (see Fig. 6).

By comparison, in the distal axial airways (G11), OVA sensitization and challenge did not affect epithelial accumulated mucosubstances. Co-sensitization with OVA plus TiO_2_ (positive control), CeO_2_ NM212, CeO_2_(0, 27 and 78% Zr), Co_3_O_4_(25% Fe_2_O_3_) and Co_3_O_4_(25% Fe_3_O_4_) NPs led to a statistically significant increase in mucosubstances compared to the OVA controls.

#### Eosinophil density

For quantification of the eosinophil density, a biomarker for an allergic airway response, lung tissue sections were stained for murine major basic protein (MBP), a specific marker for eosinophils.

##### Parenchymal lung tissue

**Fig. 7:**
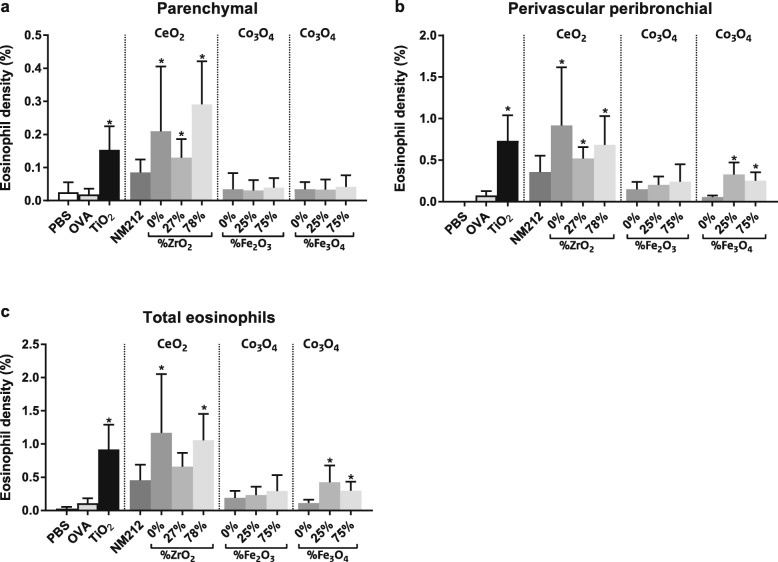
Eosinophil density. Quantification of the eosinophil density in the parenchymal (**a**), perivascular and peribronchial (**b**), and total (**c**) lung tissue. Mean ± SD, *n* = 6 except OVA where *n* = 8, * = statistically significant different from OVA controls (*p* < 0.05)

OVA sensitization and challenge did not increase the overall eosinophil density in the pulmonary parenchyma compared to non-sensitized mice (PBS controls). Co-sensitization of OVA with TiO_2_ (positive control) and CeO_2_ NPs significantly increased eosinophil density compared to OVA alone. This increase was statistically significant in all these groups, except for the OVA plus CeO_2_ NM212 NPs group. Co-sensitization of OVA with undoped and doped Co_3_O_4_ NPs had no effect on eosinophil density (Fig. 7a).

##### Perivascular and peribronchial lung tissue

OVA sensitization and challenge did not significantly affect the eosinophil density in the peribronchial and perivascular region of the lung compared to non-sensitized mice (PBS controls). Co-sensitization of OVA with TiO_2_ (positive control), CeO_2_(0, 27 and 78% Zr) and Co_3_O_4_(25 and 75% Fe_3_O_4_) NPs caused significant increases in eosinophil density compared to OVA alone (Fig. 7b).

##### Total lung

OVA sensitization and challenge did not significantly alter the overall pulmonary eosinophil density compared to non-sensitized mice (PBS controls). Co-sensitization of OVA with TiO_2_ NPs (positive control), CeO_2_(0 and 78% Zr) and Co_3_O_4_(25 and 75% Fe_3_O_4_) NPs caused significant increases in eosinophil density compared to OVA alone (Fig. 7c).

#### B-lymphocytes density

For quantification of the B-lymphocytes density lung tissue sections were stained for CD45R, a marker for B-lymphocytes.

**Fig. 8:**
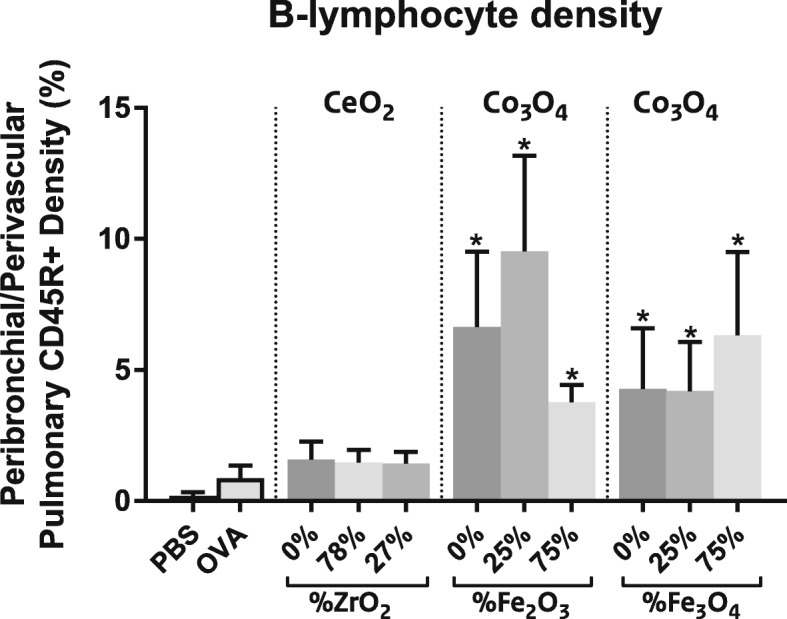
B-lymphocyte density. Quantification of the B-lymphocyte density in the perivascular and peribronchial lung tissue. Mean ± SD, *n* = 6 except OVA where *n* = 8, * = statistically significant different from OVA controls (*p* < 0.05)

Co-sensitization with OVA and Co_3_O_4_ NPs induced a severe accumulation of B-lymphocytes (CD45+ cells) in the perivascular/peribronchiolar interstitium, indicative for a chronic inflammatory response. By comparison, accumulation of B-lymphocytes in lungs from OVA plus CeO_2_ NP-sensitised mice was not as evident (Fig. 8).

### Lung histopathology

**Fig. 9:**
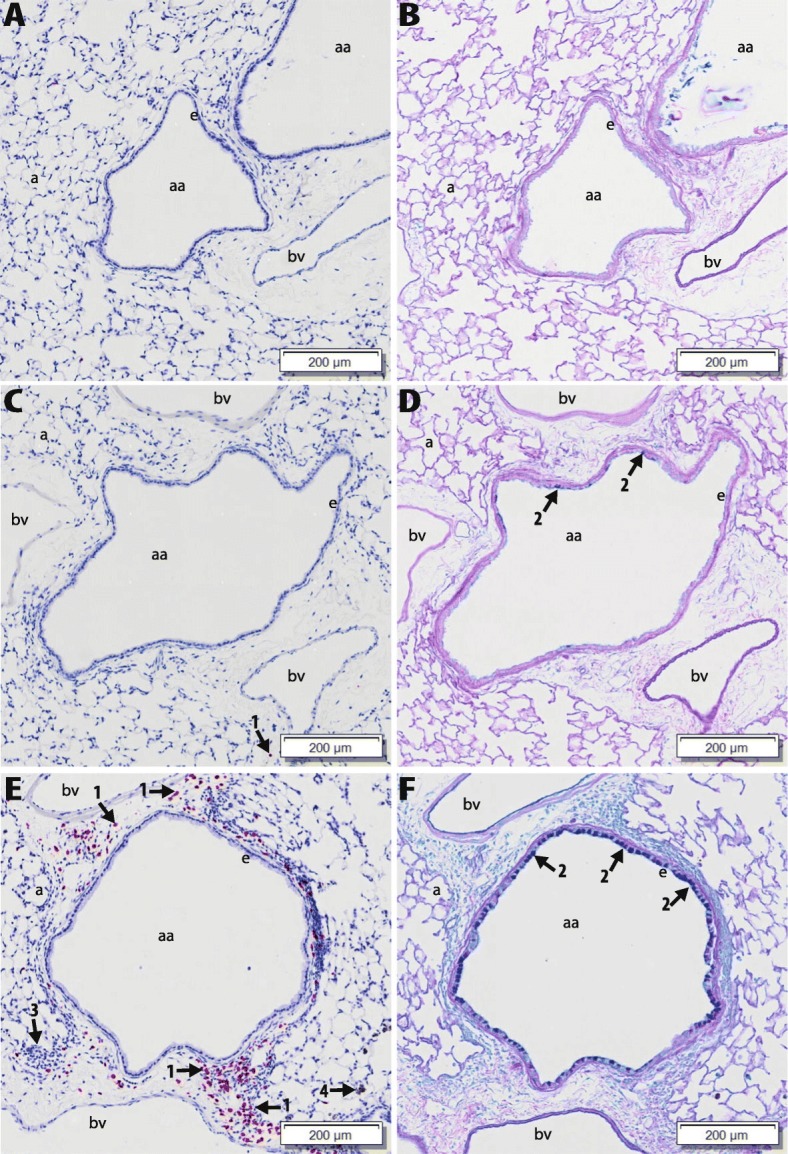
Examples of observed histopathological findings in lungs. Light photomicrographs of G11, small-diameter, distal axial airway from the lungs of PBS-treated mice (**a**, **b**), OVA-treated mice (**c**, **d**) and OVA+TiO_2_ NP treated mice (**e, f**). Lung tissue sections for **a**, **c** and **e** were stained for murine major basic protein, a specific marker for eosinophils (red chromogen-stained cells; arrow 1). Tissue sections **b**, **d** and **f** were stained with Alcian Blue/Periodic Acid Schiff (AB/PAS) for neutral and acidic mucosubstances (dark magenta stain; arrow 2) in mucous cells of the airway epithelium (e). aa: axial airway lumen; bv: blood vessel; a: alveolus; arrow 3:mononuclear cell infiltrate (mainly lymphoid); arrow 4: TiO_2_ NP-laden alveolar macrophages

**Fig. 10:**
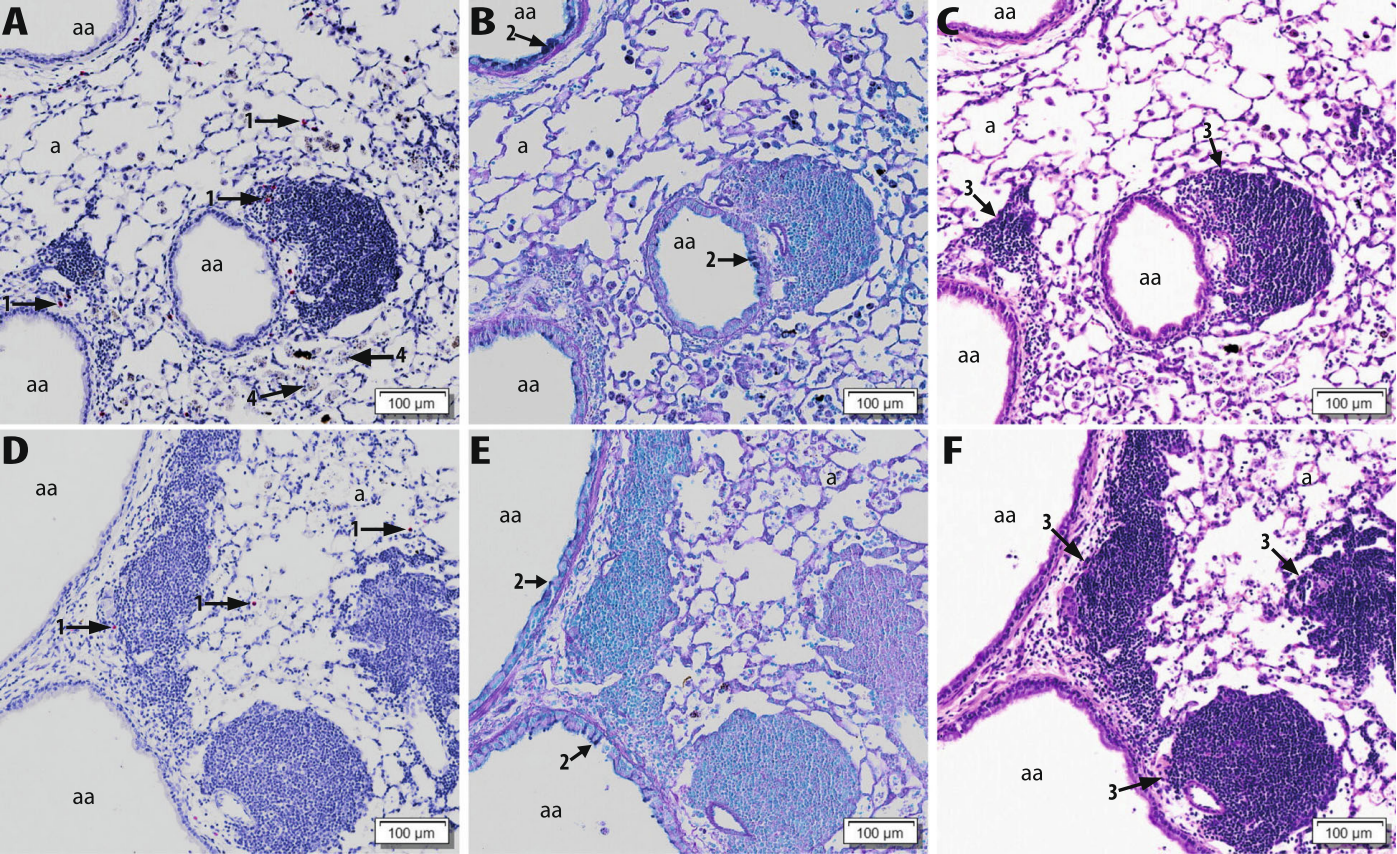
Examples of observed ectopic lymphoid structures in lungs. Light photomicrographs of G5, principally large-diameter, proximal axial airway from the lungs of OVA+Co_3_O_4_(0% Fe_2_O_3_) NP treated mice (**a**, **b**, **c**), and OVA+ Co_3_O_4_(25% Fe_2_O_3_) NP-treated mice (**d**, **e**, **f**). Lung tissue sections for A and D were stained for murine major basic protein, a specific marker for eosinophils (red chromogen-stained cells; arrow 1). Tissue sections B and E were stained with Alcian Blue/Periodic Acid Schiff (AB/PAS) for neutral and acidic mucosubstances (dark magenta stain; arrow 2) in mucous cells of the airway epithelium. Tissue sections C and F were stained with hematoxylin and eosin (H&E). arrow 3: ectopic lymphoid structures (ELS), arrow 4: Co_3_O_4_ NP-laden alveolar macrophages; aa: axial airway lumen; a: alveolus

Examples of histopathological findings in the scanned and digitized histologic slides of the lung tissue sections are shown in Figs. 9 and 10. No exposure related microscopically observable lung lesions were found in the PBS control group (Fig. 9a and b). All mice that were intranasally sensitized and challenged with OVA (OVA controls) had some degree of histopathology in the lung that was characteristic of allergic airway disease. This histopathology consisted of a mixed inflammatory cell influx containing eosinophils, lymphoid cells (lymphocytes and plasma cells) and, to a lesser extent, neutrophils in the interstitial tissue surrounding blood vessels and conducting airways (perivascular and peribronchiolar inflammation) (most conspicuous in G5, see Fig. 9c, but also in G11). Associated with these inflammatory lesions, there was mucous cell metaplasia of the airway epithelium (appearance of mucous cells with AB/PAS-stained mucosubstances) (see Fig. 9d).

Mice co-exposed to OVA and NPs had a mild to moderate multifocal accumulation of particle-laden, hypertrophic macrophages in alveolar air spaces (most conspicuous in centriacinar regions of the lung) and more severe inflammatory or epithelial lung lesions compared to mice exposed to OVA alone. Mice co-exposed to OVA and TiO_2_, CeO_2_ NM212, or Zr-doped CeO_2_ NPs had more severe lymphocytic and eosinophilic inflammation as (see Fig. 9e) as well as more severe mucous cell metaplasia (most conspicuous in G11, see Fig. 9f) compared to the OVA-alone treated mice (see Fig. 9c and d).

In contrast, OVA and Co_3_O_4_(with or without Fe-doping) co-exposed mice did not have more severe eosinophilic inflammation or mucous cell metaplasia as compared to OVA-alone treated mice. Interestingly, these OVA and Co_3_O_4_ co-exposed mice exhibited more severe perivascular or peribronchiolar lymphoid cell accumulation compared to mice co-exposed to the other NPs, organized in ectopic lymphoid-like structures (ELS, see Fig. 10a, b and c). ELS are tertiary lymphoid organs, associated for example with infections and autoimmune diseases, that develop in areas of chronic inflammation and are characterized by the formation of organized B- and T-cell aggregates and germinal centres with follicular dendritic cell networks [23, 24]. These lung lesions were also observed in mice co-exposed to OVA and Co_3_O_4_ with Fe-doping (using both Fe_2_O_3_ and Fe_3_O_4,_ see Fig. 10d, e and f).

## Discussion

All NPs investigated possessed adjuvant activity, leading to changes in OVA-specific IgE and IgG1 plasma levels, differential cell count and cytokines in BALF, and histopathological detection of mucosa cell metaplasia and eosinophil density in the conducting airways. Co-exposure of OVA and Co_3_O_4_ NPs also induced perivascular and peribronchiolar lymphoid cell accumulation and the formation of ELS in lungs.

### Adjuvant activity

Although the increased OVA-specific IgE and IgG1 plasma levels of all OVA + NP exposed groups compared to OVA controls were not statistically significant for all NPs, they do indicate that NPs have the capacity to enhance allergic sensitisation in mice. This is supported by the increases in eosinophils, lymphocytes, IL-4, IL-5 and IL-6 in BALF after co-exposure of OVA and many NPs compared to the OVA controls. In our previous study, we found that SiO_2_ and TiO_2_ NP exposure alone (without OVA) during the induction phase did not lead to an increase in OVA-specific IgE and IgG1 plasma levels or eosinophils in BALF after OVA challenge [9, 10]. These findings suggest that the allergic response can only be enhanced via co-exposure of the allergen and NPs, and not by exposure to NPs alone. In the current study, all NPs were only tested in combination with OVA, and the results show that all NPs were able to act as adjuvant, albeit to varying degrees.

### The influence of the chemical composition

**Fig. 11:**
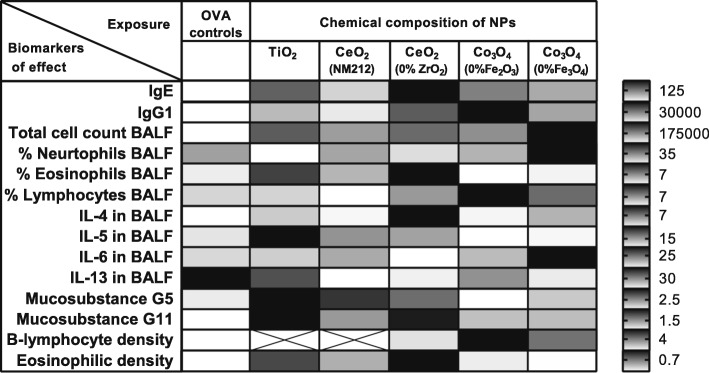
The influence of the chemical composition on the different biomarkers of effect. The shading indicates the lowest (light grey), intermediate (medium grey) or highest (dark grey) response of an NP (relative to the OVA controls) for each of the biomarkers. The bar on the right side shows for each row the value corresponding with the indicated (medium) grey shading.

The differences in OVA-specific IgE and IgG1 plasma levels of the animals co-sensitized to OVA and the different NPs indicate that the chemical composition of the NPs influences their potency to exacerbate allergic airway sensitization. The relative adjuvant potencies of the different NPs varied with the different biomarkers of effects (Fig. 11), indicating that the chemical composition also influences the type of the allergic response.

#### Type of the immune responses

Animals co-exposed to OVA and undoped CeO_2_ NPs showed higher OVA-specific IgE plasma levels compared to animals co-exposed to OVA and the other undoped NPs (TiO_2_ NPs, CeO_2_ NM212 NPs, and Co_3_O_4_ NPs), indicative for a type 1 (immediate or IgE mediated) allergic response. Additionally, indications for a Th2 type immune response were observed, characterised by an increase in the percentage of eosinophils and increased concentrations of the Th2 cytokines IL-4 and IL-5 (but not IL-13) in the BALF. Animals co-exposed to OVA and the OECD representative manufactured nanomaterial CeO_2_ (NM212), also showed some indications of a Th2 type response, albeit less marked than those observed in animals co-exposed to OVA and CeO_2_(0% Zr) NPs: plasma OVA-specific IgE levels, percentage of eosinophils in BALF and BALF IL-4 concentrations were much lower in animals co-exposed to OVA and CeO_2_ NM212 than in animals co-exposed to OVA and CeO_2_(0% Zr) NPs. In contrast, concentrations of IL-5 in BALF were similarly affected by both forms of CeO_2_. These findings are in line with a previous in vivo study in which CeO_2_ NPs induced increased antigen specific IgE levels, eosinophils, Th2 cytokines and Th2 chemokines when co-exposed with house dust mite [25] and an in vitro study in which CeO_2_ NPs exposure of human dendritic cells induced a Th2-dominated cell profile [26].

Co-exposure to OVA and undoped Co_3_O_4_ NPs also showed responses indicative of a type 1 allergic response, albeit, the OVA-specific IgE plasma levels were lower compared to co-exposure to OVA and the CeO_2_(0% Zr) NPs. Furthermore, the response after co-exposure to OVA and Co_3_O_4_ NPs was characterised by no or less marked increases in in BALF IL-4 and IL-5 concentrations and the percentage of eosinophils in BALF, but a more pronounced increase in the BALF IL-6 concentration and the percentage of lymphocytes in BALF compared to co-exposure to OVA and the CeO_2_(0% Zr) NPs. This pattern of response is in line with a previous in vivo study in C57Bl/6 mice in which subcutaneous administration of Co_3_O_4_ NPs with OVA induced both Th1 and Th2 type responses [27]. Several biomarkers, including the OVA-specific IgG1 plasma levels and IL-6 concentrations in the BALF, were remarkably different in animals co-exposed to OVA and the two different types of undoped Co_3_O_4_ NPs, indicating that not only chemical composition, but also other physicochemical characteristics of the NPs are of influence to the allergic response. Although, the production process of the two undoped Co_3_O_4_ NPs was the same, they differed in the degree of crystallinity, aggregation, Co/Fe ratio and spatial distributions of the metals in the NPs [18].

The immune response after co-exposure to OVA and TiO_2_ NPs was indicative for a Th2 immune response, characterised by an increase in eosinophils, IL-4 and IL-5 in BALF. Previous studies with TiO_2_ NPs have found contradictory results regarding effects on immune responses. Schanen et al. [26] found that exposure of dendritic cells to TiO_2_ NPs led towards Th1-based responses, while Vandebriel et al. [10] and de Haar et al. [28] observed Th2 type immune responses in mice after co-exposure to OVA plus TiO_2_ NPs. However, the contradictory result may have been caused by differences in the physicochemical characteristics (e.g. sizes and/or crystallinity) of the TiO_2_ NPs used in the studies by Schanen et al. [26] and de Haar et al. [28], compared to the TiO_2_ NPs used in our study, which were the same TiO_2_ NPs as Vandebriel et al. [10]. Although this study adds weight to the steering of the immune response towards a Th2 response by TiO_2_ NP co-exposure with antigens, more studies which measure a wide range of Th2 and Th1/Th17 associated biomarkers are needed to gain more insight in the type of adjuvant activity associated with by TiO_2_ NPs exposure.

#### Lung morphology and ectopic lymphoid-like structures

Animals co-exposed to OVA and TiO_2_, CeO_2_ NM212, or CeO_2_(0% Zr) NPs had more severe mucous cell metaplasia (most conspicuous in G11, but also in G5), as well as more severe eosinophilic inflammation, compared to the OVA-alone treated mice. OVA and Co_3_O_4_ co-exposed mice showed more severe perivascular and peribronchiolar lymphoid cell accumulation, organized into ELS. These findings were accompanied by an increase in lymphocytes in BALF. In mice broncho-associated lymphoid tissue (BALT) is usually absent, however, it can be induced (inducible BALT or iBALT) by inflammatory processes and infections [29]. In humans, iBALT formation can be observed in the lung in response to various types of infectious and inflammatory states, caused by infectious organisms, diesel exhaust, cigarette smoke silica, and various autoimmune diseases [30, 31]. In previous studies lymphocyte foci or aggregates have been observed in lungs of rats after intratracheal instillation of Co_3_O_4_ NPs [32], as well as in lungs of mice after intranasal exposure to OVA [33], crystalline silica particles (1.5–2.0 μm) [34] and after inhalation of CdO NPs [35]. However, the role of iBALT in the pathophysiology of chronic allergic diseases, such as asthma, is poorly understood [30, 31]. Studies investigating whether or not iBALT is involved in the development or progression of allergy or asthma showed conflicting results and the pathways that control the development and function of iBALT are poorly understood.

### The influence of redox modification by doping

The differences in immune responses observed between the groups exposed to the same type of NPs, but different amounts of doping (Zr-doping for CeO_2_ NPs and Fe-doping for Co_3_O_4_ NPs), indicate that redox modification can influence the adjuvant activity of NPs. However, the amount of doping did not always show a clear trend in the response of the different biomarkers of effects. Some biomarkers indicated that the undoped NPs exhibit most adjuvant activity, while other biomarkers indicated that either the NP with the lowest amount of doping or the NPs with the highest amount of doping exhibited most adjuvant activity. Additionally, most biomarkers of effects neither decreased nor increased with greater degrees of doping (see in Additional file 3).

This lack of trend between increasing amounts of doping and the biomarkers of effects is also observed in the EPR data, in which only the Co_3_O_4_(0, 25 and 75% Fe_3_O_4_) NPs showed a decreasing scavenging capacity with increasing amounts of doping (see Fig. 2). This decrease in free hydroxyl radical scavenging capacity was unexpected based on the Ec levels of Co_3_O_4_ and Fe_3_O_4_. We expected that doping using Fe_3_O_4_ would increase, rather than decrease the ability of Co_3_O_4_ to scavenge free hydroxyl radicals, because the Ec level of Co_3_O_4_ overlaps with the cellular redox potential, whereas that of Fe_3_O_4_ does not. On the other hand, the superoxide generation did increase with Fe-doping of Co_3_O_4_ NPs using 25% Fe_3_O_4_, but this ROS generation was not further increased with increasing amounts of Fe-doping. It is possible that differences in particle size and aggregation counterbalanced any doping-related differences in free radical generation or scavenging [11, 36], as the particle size in water as measured with disc centrifuge was larger for the Co_3_O_4_(75% Fe_3_O_4_) NPs compared to Co_3_O_4_(25% Fe_3_O_4_) NPs, although the difference in particle size between these two NPs was relatively small. Nonetheless, we would still have expected more notable differences in free radical generation from the surfaces of the differently doped materials. Physicochemical characterisation of the materials indicates successful incorporation of the doping into the crystal structure of the NP [18], thus at present we can only speculate as to the reason for the limited effect of doping on free radical generation.

Since, the amount of doping or band gap energy of the NPs were not predictive for adjuvant activity or the ability to generate or scavenge specific radical, we also investigated if the ability of the NPs to generate or scavenge specific radicals was predictive for the adjuvant activity.

**Fig. 12:**
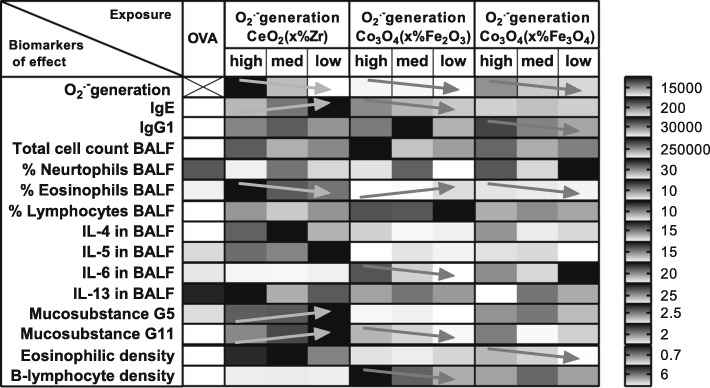
The influence of the ability of NPs to induce superoxide generation as measured in an acellular EPR assay on the different biomarkers of effects. The shading indicates the lowest (light grey) to highest (dark grey) response of an NP (relative to each other) for each of the biomarkers. The arrows indicate an increase (↗) or decrease (↘) with decreasing amount of superoxide generation. The bar on the right side shows for each row the value corresponding with the indicated (medium) grey shading.

The superoxide generation as measured with the EPR was not predictive for the adjuvant activity. Only a few biomarkers of effects decreased with decreasing amounts of superoxide generation and these biomarkers of effects were different for each of the NP series (see arrows in Fig. 12).

**Fig. 13:**
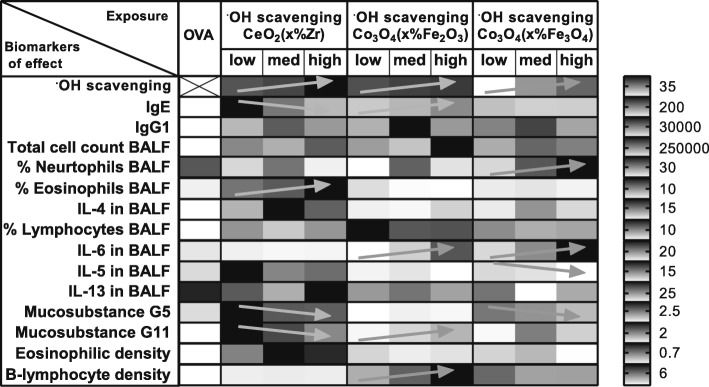
The influence of the scavenging capacity of the NPs as measured in an acellular EPR assay on the different biomarkers of effects. The shading indicates the lowest (light grey) to highest (dark grey) response of an NP (relative to each other) for each of the biomarkers. The arrows indicate an increase (↗) or decrease (↘) with increasing scavenging capacity. The bar on the right side shows for each row the value corresponding with the indicated (medium) grey shading.

The scavenging capacity as measured with the EPR was also not predictive for the adjuvant activity. Only a few biomarkers of effects decreased with increasing amounts of scavenging capacity and these biomarkers of effects were different for each of the NP series (see arrows in Fig. 13).

While EPR is a useful technique to predict the ability of NPs to generate cellular oxidative stress, this acellular approach provides only a facet of the redox reactivity of NPs within cellular compartments. However, due to the number of NPs under investigation, a comprehensive comparison of redox activity in cellular assays was not possible within the current study. Therefore, a possible explanation for the lack of trend between the EPR results and the adjuvant activity is that redox activity of the NPs may change due to interaction with the physiological environment, e.g. by corona formation, aggregation, elimination, redox reactions, etc. Interactions with the physiological environment are known to influence the reactivity, cellular uptake and toxicity of the NPs [37–40]. Han et al. [41] found that co-exposure of OVA and polyethylene glycol-conjugated (PEGylated) SiO_2_ NPs induced less severe airway inflammation compared to non-coated SiO_2_ NPs. It was suggested that this was due to an increased aggregation of the PEGylated SiO_2_ NPs in saline compared to the non-coated SiO_2_. However, the small differences in the (aggregated) particle diameter of the NPs in water observed in our study (see Table 2) would be very unlikely to significantly influence adjuvant potency. Larger differences in the (aggregated) particle diameter of the NPs were observed in a more physiologically relevant environment (i.e. buffered cell culture medium with proteins from fetal calf serum) (see Table 2). However, no relation between these differences in aggregated size and the adjuvant potencies of the different NPs found. Horie et al. [42] found differences in OVA-specific IgE and IgG1 concentrations in the blood of mice exposed to ZnO compared to SiO_2_-coated ZnO NPs after subsequent exposure to OVA. The investigators suggested that these differences might be due to differences in solubility and continuous Zn^2+^ release from the NPs. Although the NPs in our study are expected to be relatively insoluble, further evaluation of the possible degradation and ion release under various physiological conditions (e.g. in lysosomal fluid) are needed to rule out this possibility. Further research is needed to better understand the mechanisms of the adjuvant activity of NPs, including the influence of the NP properties and NP interactions with their physiological environment, including allergens (such as OVA), on the adjuvant activity.

## Conclusions

The chemical composition of NPs in this study influenced both their relative potency to exacerbate allergic airway sensitization to ovalbumin (OVA), and the type of the immune response. The adjuvant activity of CeO_2_ NPs seems to be primarily mediated via a Th2-type immune response, whereas the adjuvant activity of Co_3_O_4_ NPs was characterised by no or less marked increases in OVA-specific IgE plasma levels, BALF IL-4 and IL-5 concentrations and percentages of eosinophils in BALF and more pronounced increases in the BALF IL-6 concentrations and percentages of lymphocytes in BALF. Co-exposure to OVA and Co_3_O_4_ NPs also induced lymphoid cell accumulation and the formation of ectopic lymphoid tissues in the lungs. No relation between the acellular redox potential and the observed adjuvant activity of the different of NPs was found, suggesting that acellular ROS assays may not be suitable to predict adjuvant capacity. These findings highlight the complexity of biological interplay between different NP species and the need for additional data and methods to formulate scenarios that better predict the adjuvant activity of nanomaterial families.

## Supplementary information

**Additional file 1.** Total and differential cell counts in bronchoalveolar lavage fluid.

**Additional file 2.** Cytokines in bronchoalveolar lavage fluid.

**Additional file 3: ** The influence of the ability of NPs to induce superoxide generation as measured in an acellular EPR assay on the different biomarkers of effects.

## Data Availability

The datasets used and/or analysed during the current study are available from the corresponding author on reasonable request.

## Abbreviations

ANOVA: Analysis of variance
BALF: Bronchoalveolar Lavage Fluid
DMPO: 5,5-dimethyl-1-pyrroline N-oxide
Ec: Conduction band energy level
EPR: Electron Paramagnetic Resonance
LAL: Limulus Amoebocyte Lysate
MBP: Major Basic Protein
NP: Nanoparticle
OVA: Ovalbumin
PBS: Phosphate-Buffered Saline
ROS: Reactive Oxygen Species
RT: Room Temperature
SD: Standard Deviation
STEM: Scanning Transmission Electron Microscopy
