# Supplementary material for: Role of chemical composition and redox modification of poorly soluble nanomaterials on their ability to enhance allergic airway sensitisation in mice
Source: Part Fibre Toxicol. 2019 Oct 28;16:39. doi: 10.1186/s12989-019-0320-6 (PMC6819391; doi:10.1186/s12989-019-0320-6)
Supplement: Supplementary file 3 — Additional file 3: The influence of the ability of NPs to induce superoxide generation as measured in an acellular EPR assay on the different biomarkers of effects. [file 12989_2019_320_MOESM3_ESM.pdf]

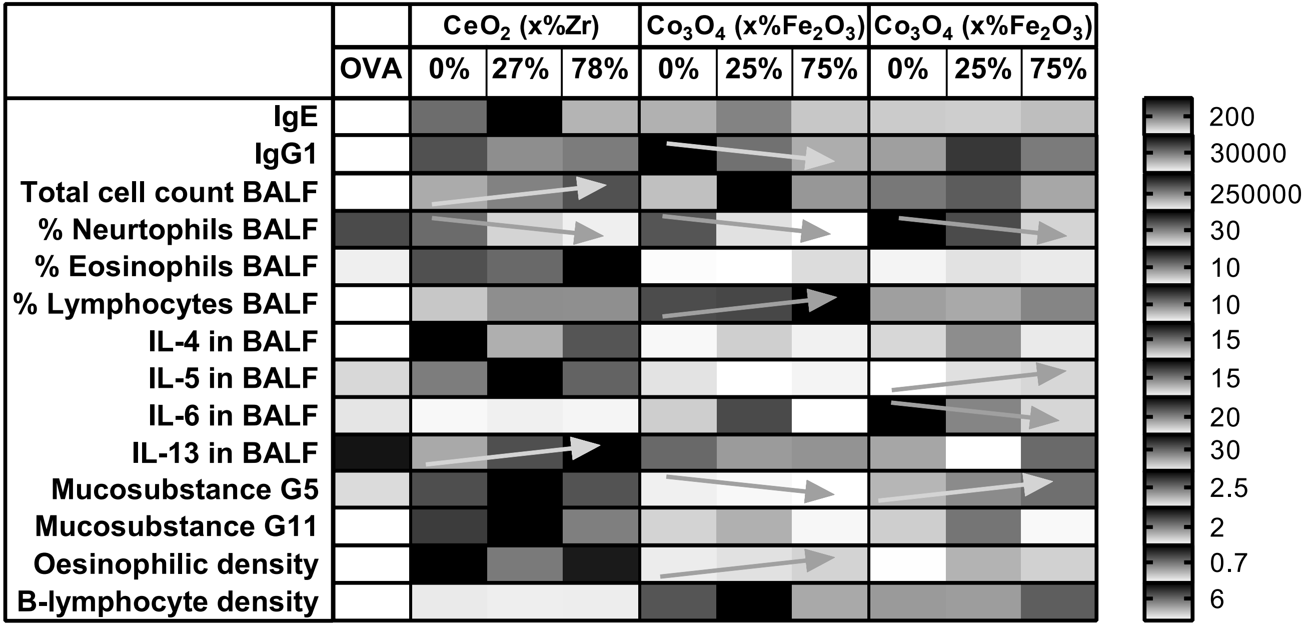

Figure S1: The influence of the ability of NPs to induce superoxide generation as measured in an acellular EPR assay on the different biomarkers of effects. The shading indicates the lowest (light grey) to highest (dark grey) response of an NP (relative to each other) for each of the biomarkers. The arrows indicate an increase (⬀) or decrease (⬂) with decreasing amount of superoxide generation.
